# A generalist institutional data repository offering both open and restricted access to support NIH data sharing compliance

**DOI:** 10.5195/jmla.2026.2339

**Published:** 2026-04-01

**Authors:** Seonyoung Kim, Xing Jian, Marcy L. Vana

**Affiliations:** 1 seonyoung.kim@wustl.edu, Senior Support Scientist, Data Management and Sharing Services Group, Bernard Becker Medical Library, Washington University in St. Louis, MO; 2 jianx@wustl.edu, Senior Support Scientist, Data Management and Sharing Services Group, Bernard Becker Medical Library, Washington University School of Medicine in St. Louis, MO; 3 vanam@wustl.edu, Associate Director, Research Services, Bernard Becker Medical Library, Washington University in St. Louis, MO

**Keywords:** Data Repository, Data Sharing, Data Curation, NIH Data Management and Sharing Policy, Institutional Repositories, FAIR data principles, Restricted Access, Open Access

## Abstract

In response to the 2023 NIH Data Management and Sharing (DMS) Policy, Washington University School of Medicine in St. Louis launched Digital Commons Data@Becker, a generalist institutional data repository supporting both open and restricted access to research data. Managed by Bernard Becker Medical Library's DMS Team, the repository offers a fully mediated curation workflow that guides researchers through consultation, metadata capture, documentation, and quality control. Draft Digital Object Identifiers (DOIs) can be issued once access type is determined, with final DOI publication following curation and QC. Restricted datasets require Human Research Protection Office (HRPO) review and Data Transfer and Use Agreements (DTUAs), while open access datasets are freely downloadable.

The repository leverages persistent identifiers such as Open Researcher and Contributor ID (ORCID iDs), Research Organization Registry (ROR) IDs, and DOIs, along with the DataCite metadata schema and custom metadata fields. Since its launch in 2023, Digital Commons Data@Becker has published 30 datasets spanning biomedical imaging, sequencing, quantitative assays, flow cytometry, and qualitative survey data. Across all datasets, there have been 4,409 views and 4,120 files downloaded, with restricted datasets generating 13 access requests, three of which were granted through DTUAs. Researchers emphasize the value of free institutional curation, flexible access models, and rapid DOI assignment.

Digital Commons Data@Becker demonstrates how a generalist institutional data repository can balance accessibility and security to support NIH compliance, while advancing FAIR (Findable, Accessible, Interoperable, Reusable) data sharing and long-term stewardship.

In response to the 2023 NIH Data Management and Sharing (DMS) Policy, Washington University School of Medicine in St. Louis (WashU Medicine) launched Digital Commons Data@Becker, a generalist institutional data repository that supports both open and restricted access to research data. Managed by Bernard Becker Medical Library’s DMS Team, the repository provides WashU Medicine researchers with a curated and FAIR (Findable, Accessible, Interoperable, and Reusable) data-sharing solution tailored to National Institutes of Health (NIH) and publisher requirements.

## PROJECT IMPLEMENTATION

The repository was deployed in early 2023, and is an instance of Digital Commons Data, the institutional version of Mendeley Data. Deposits are mediated entirely by the Becker DMS Team to improve metadata quality, ensure compliance with human participant data requirements, and streamline workflows for researchers. Submissions begin with an online consultation request, followed by individualized consultations. The curation workflow includes identifying data types, collecting required metadata and supporting documents, converting files into open formats, and determining access type (open or restricted). A draft DOI can be issued once access type is determined, typically within a day for open access datasets and within a week for restricted access datasets. After curation and quality control, the final dataset with DOI is usually published in coordination with manuscript acceptance or publication, ensuring timely and reliable data availability.

Datasets resulting from research with human participants undergo review with the WashU Human Research Protection Office before access type is finalized. Access requests to published datasets with restricted access are handled through a Data Transfer and Use Agreement (DTUA) process requiring institutional signature and involving WashU’s Joint Research Office for Contracts. In contrast, datasets published with open access can be freely accessed and downloaded.

## STAFF INVOLVEMENT

The project is led by the Becker DMS Team (two data curators: Seonyoung Kim, PhD, Xing Jian, PhD; and repository manager Marcy Vana, PhD, who performs the final QC). The Becker DMS Team curates deposited datasets, develops policies, creates FAQs, documentation, and training resources, and provides ongoing feedback to the Digital Commons Data support team to enhance functionality.

## TECHNOLOGIES USED

Digital Commons Data@Becker leverages persistent unique identifiers such as Open Researcher and Contributor ID (ORCID iDs), Research Organization Registry (ROR) IDs, and Digital Object Identifiers (DOIs), and uses the DataCite metadata schema. A built-in version comparison tool highlights changes across revisions using color code, while custom metadata fields such as keywords, other contributing organizations (without ROR IDs), and a revision history field, enhance dataset description. Built-in metrics track views and downloads for open access datasets but only track views for restricted access datasets. Download metrics of restricted access datasets are tracked manually based on the DTUA process records. The repository is registered with FAIRsharing and re3data, increasing visibility and trust.

## OUTCOMES AND ASSESSMENT

Since launch, the repository has supported deposits spanning biomedical imaging, sequencing, quantitative assay results, flow cytometry, and qualitative survey data. As of September 2025, 30 datasets have been published (19 open access, 11 restricted). Collectively, these datasets have received 4,409 views and 4,120 files have been downloaded. For restricted datasets, 13 requests were submitted and three granted after DTUA execution. Researchers have emphasized the value of free expert curation, flexible access options, and rapid DOI assignment. The flexible open and restricted access options reduce reliance on costly external repositories and allow WashU Medicine investigators to retain stewardship of sensitive data while meeting funder expectations. It also enables linked deposits of mixed datasets, such as openly available survey instruments paired with de-identified survey results under restricted access (e.g., https://doi.org/10.17632/6z94cbyt2r.1 and https://doi.org/10.17632/n8r43gf2dm.1), lowering barriers for discovery while protecting sensitive information.

## CONCLUSION

Digital Commons Data@Becker demonstrates how a generalist institutional data repository can balance accessibility and security to support NIH DMS policy compliance. By offering both open and restricted access, coupled with a fully mediated expert curation workflow, the repository ensures high-quality, FAIR-aligned data sharing that empowers researchers and strengthens institutional support for research transparency.

## REPOSITORY ACCESS

https://digitalcommonsdata.wustl.edu/research-data

